# Evaluating the Coordinated Development between Urban Greening and Economic Growth in Chinese Cities during 2005 to 2019

**DOI:** 10.3390/ijerph19159596

**Published:** 2022-08-04

**Authors:** Zhen Yang, Weijun Gao

**Affiliations:** 1 College of Civil Engineering and Architecture, Weifang University, Weifang 261061, China; 2 Innovation Center for CIM + Urban Regeneration, Qingdao University of Technology, Qingdao 266033, China; 3 Faculty of Environmental Engineering, The University of Kitakyushu, Kitakyushu 808-0135, Japan; 4 iSMART, Qingdao University of Technology, Qingdao 266033, China

**Keywords:** urban greenspace, economic performance, coupling coordination model, spatio-temporal changes, cluster analysis

## Abstract

Balancing economic growth with environmental protection is vital for the sustainable development of cities and regions. However, urban greening has rarely been considered in extensive studies. This study incorporates urban greening into a coupling coordination degree (CCD) model, in order to evaluate its coordination with economic performance. A total of 286 cities in China between 2005 and 2019 were selected as specific study subjects. Meanwhile, clustering method was used to classify different clusters based on CCD values, the Gini coefficient analysis was applied to discover the CCD values inequality characteristics and the exploratory spatial data analysis (ESDA) method was employed to study the CCD values spatial aggregation features. The results indicate that the CCD values presented significant spatial heterogeneity. Spatially, the CCD values were divided into eight clusters, with those in the eastern region generally being higher than in the central and western regions. Temporally, the CCD in all cities showed an increasing trend, but more than 60% of cities were still in the uncoordinated or low-level coordination stage. In addition, inequality and spatial aggregation characteristics were observed in CCD values, both of which presented decreasing trends. Greening has a stronger influence on the linked and coordinated growth of the two systems; therefore, we propose policy recommendations for pursuing the development of environmentally friendly cities from different aspects. In summary, this research allows for a better understanding of economic and environmental relationships, thus contributing to the objective of creating sustainable cities and communities.

## 1. Introduction

The rapid expansion of cities and economic growth on a global scale since the beginning of the 21st century has been accompanied by the emergence of a wide range of environmental problems, including environmental degradation, ecological damage, global warming, air and water pollution and loss of biodiversity [1,2,3,4]. Understanding the interplay between the economy and the environment is essential for long-term growth in the regions. At the same time, the wide range of environmental repercussions makes striking a balance between the economy and the environment a difficult task. There has already been a substantial amount of work investigating the interactions between these two systems, either beginning with carbon emissions or with the relationship between economic growth and PM_2.5_ [5,6,7,8,9,10,11]. These studies have provided reliable and detailed insights into the impact of human economic activity on urban environmental systems; however, given the wide range of environmental consequences, more components of the urban environment must be included in economic environmental sustainability assessment frameworks [12,13], which will allow us to better comprehend the connected urban-environmental system and hence contributing to their long-term sustainability.

Urban greening has rarely been taken into account in the extensive efforts to study the economic–environmental balance. Broadly speaking, urban greening refers to the planting of trees, flowers, and grasses to cover or decorate a certain area of ground (space) in a city. In this study, we consider urban greening in terms of urban public green spaces (UPGS), including parks, gardens, street plantations, lawns, crops, and forests, as well as artificial green spaces (e.g., roadside green, riverfront green, green spaces around housing, green spaces around institutions, places and squares), which are intricate and varied elements of the urban ecosystem [14,15]. On one hand, as one of the most important elements of the urban environment, UPGSs have the ability to drastically alleviate a wide range of urban problems, including regulating the urban reducing the impact of climate and mitigating the urban heat island effect, absorbing particulate air pollutants, infiltrating storms, and reducing noise levels [16,17,18,19,20]. On the other hand, they also play a vital role in enhancing urban living quality [21,22]. As important locations for living and social interactions, UPGSs have a tremendous impact on the physical and emotional well-being of residents, not only indirectly reducing all-cause mortality and the risk of chronic diseases, but also promoting mental health, reducing stress, and contributing to self-recovery [23,24,25,26,27,28].

In the context of rapid economic development, it is essential to quantify the equilibrium between these two systems in order to create credible knowledge to achieve the goal of a more pleasant and sustainable urban environment in the future. Most previous investigations involving urban greening have mostly looked at the economic impact of greening and broad evaluation of the quality of urban greening. Specifically, socio-economic attributes, such as gross regional product per capita, percentage of industry, built-up area, urbanization rate, and population density, have a potential impact on urban greening; however, whether this correlation is positive or negative is still a matter of debate in academic circles [29,30,31,32,33]. The other side of the coin is the quantification of accessibility and equity of access to urban green spaces [15,34,35,36,37,38,39]. Nevertheless, past research has only looked at multiple viewpoints on the single systems individually, rather than focusing on the balance between the two systems. More recently, the CCD model has become popular for evaluating the extent of cooperation between two or more systems [40,41,42]. It was created using coupling theory, which describes how different systems interact. In contrast to standard coupling analysis methodologies, the CCD model highlights the relevance of multidimensional sustainability through cross-system coordination [43]. The CCD model, in particular, may provide a coordination attribute to each city in each year, allowing for a more in-depth examination of the spatio-temporal dynamics of coordination through a geographical approach [40,44]. As a result, the pattern of urban greening during periods of strong economic expansion may be studied in both temporal and geographical aspects. Therefore, in this study, we innovatively include urban greening in a cross-system sustainability assessment framework, which investigates the coupled and coordinated development of urban greening and economic growth by focusing on the balance between them on the one hand, and complements studies on sustainable cities and communities on the other hand, laying the groundwork for future analysis of the coordination of urban economy and urban environment in multiple urbanization scenarios.

Due to disparities in economic growth, as well as the considerable variety of the natural environment, the relationship between the economy and urban greening may present substantial temporal and geographical fluctuations. Therefore, there is a need to further investigation of the regional inequalities in the coordination of the two systems, as well as the temporal differences in spatial aggregation. Inequalities in economic growth and patterns of agglomeration have been extensively verified worldwide [3,45]. Due to various urbanization processes, diverse urban planning and construction philosophies, and unique geographical contexts, the accompanying urban greening is also heterogeneous in time and space [46,47,48,49,50]. In summary, both economic growth and urban greening should be characterized in terms of regional inequalities and spatial aggregation. Does such a pattern of coordination between the two systems exist? If so, how has the coordination between them evolved?

Based on the above context, this research has two major goals: (1) To include the spatio-temporal dynamics of urban greening into a cross-system coupled analytical framework, in order to determine how well it coordinates with economic development; (2) based on the findings of the coordinated evaluation analysis, we aim to suggest a policy framework for achieving a sustainable urban environment in the future, with regard to new urbanism. Therefore, we specifically conduct analyses in the following ways: (1) The CCD model is used to quantify the urban economy and urban greening using two indicators-gross domestic product (GDP) and public green area per capita, respectively, in order to assess the coordination between the two systems. The higher the coordination degree, the more sustainable the urban development; (2) the spatio-temporal patterns of the two systems and CCD are studied using the long time-series clustering approach, in order to investigate the spatio-temporal non-stationarity of the indicators; (3) we focus on district inequality and CCD spatial aggregation, as well as variations in these phenomena; and (4) policies and practice for achieving a more environmentally friendly urban environment in the new era are explored. The remainder of this paper is structured as follows: the literature review is presented in Section 2, and the materials and methods are outlined in Section 3, analytical findings and discussion are presented in Section 4, and our conclusions are provided in Section 5.

## 2. Literature Review

**Figure 1:**
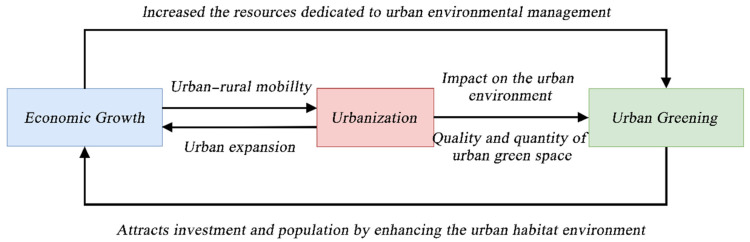
Relationships between economic growth, urbanization, and urban greening.

In fact, economic growth and urban greening are inter-related components, as shown in Figure 1. Scholars generally believe that the geographical and temporal dynamics of urban greening are intimately tied to urban development, rather than direct economic growth and social transformation [51], that is to say, urbanization is the key to linking economic growth and urban greening. First, economic growth and urbanization have a long-term bi-directional causality: economic growth enhances urban incomes and social welfare, and as a key driver, it necessarily enables rural–urban migration, resulting in extensive urban expansion [52,53]. Conversely, as a quick and efficient means of increasing municipal revenues, urban expansion based on land finance has made an outstanding contribution to economic growth, which has been particularly evident in China’s development over the last decade or so [53]. Secondly, rapid urbanization and uncontrolled urban sprawl have dramatically changed land-cover and land-use types [54,55,56], and shift the from green space to built-up land has significantly altered the quantity and quality of urban green spaces, a process that is prevalent in cities around the world, particularly in Asia and Oceania [15,57,58]. For example, between 2000 and 2014, a study of 90 major Chinese cities found a considerable decrease in urban green cover of all urban areas in the aforementioned cities [59,60]. Furthermore, economic growth contributes to greening levels in cities; specifically, economic prosperity provides cities with more financial resources, including those targeted at urban greening management. As a result, wealthier cities tend to manage urban greening in an efficient and timely manner [6,61]. Finally, urban greening improves the city’s living environment, attracting not only more external capital for investment, but also a great number of foreigners to establish themselves in the city, providing an abundance of labor resources to ensure rapid economic development [24,49,62,63].

Today, green space and green infrastructure expansions are rather effective tools for economic development, tourism attraction, and neighborhood revitalization, especially so when new businesses open up in the vicinity of a new green amenity [64]. The desirability of a neighborhood for real estate investors and residents is often enhanced when it becomes greener, which eventually contributes to higher property values [65]. Research on real estate indeed reveals that urban green infrastructure positively influences home prices. For example, a synthesis of many studies showed that for every 1% increase in the area of green open space in a neighborhood, it translated into a 2.25% increase in value as measured through willingness to pay; and the value of an average green open space was $1550 per hectare per year (in 2003 US dollars). For example, in Philadelphia, the redevelopment of the Delaware River waterfront involved the creation of the quasi-public Delaware River Waterfront Corporation in 2009, which used expected increases in real estate values and associated tax revenues near the park as a key justification for public expenditure on greening, thereby attaching greening to economic development benefits. In Mississauga, the addition of several downtown parks, green streets, and green connections has been a strategy to bring activity back into the downtown, and to eventually attract sustained higher level socio-economic standing for residents by creating a network of “great people places”. Between 1990 and 2010, downtown Mississauga received several green amenities, and after the release of the 2010 Downtown 21 Master Plan, the municipality announced several new parks, green streets, and green connections. Among others, it constructed the Scholars’ Green downtown park at Sheridan College as an “outdoor living room”.

The “greening” strategy is increasingly becoming one of the core strategies for urban development, especially in many cities in Europe and North America. We focused on the greening governance strategies of the 99 sample cities in these regions and analyzed the four different dominant policy styles that emerged, namely, the level of greening integration, the level of greening rhetoric, the level of greening implementation, and the level of greening participation. Green space is not a niche theme in cities with highly integrated greening policies, but has been a key strategic project throughout its geographic scope for many years and has been mainstreamed into different urban policies, including urban regeneration, housing or urban health. Cities that focus on the rhetorical level of greening in presenting their identity to the world are more focused on highlighting how greening permeates the culture of the city and its visionary processes (i.e., green discourse does not always reflect greening actions on the ground), and such cities include Vancouver, Cleveland, and Copenhagen, which aim to be recognized as global leaders in green urban planning. Some cities have implemented more greening projects in their urban landscapes than others. Examples include parks, open natural spaces, picturesque waterfronts, and urban greenways; such cities include Louisville, Kansas City, Seville, and Stockholm. Actively building and opening up green spaces to make them successful is often more helpful in furthering greening policies. In the larger number of cities surveyed, procedural involvement of residents in the visioning, planning, and management of green spaces has been key to greening policy. Whether through municipally funded participation to guide greening goals and outcomes or through the transfer of some form of direct control over greening initiatives, these cities have harnessed the creativity, neighborhood experience and self-organization of de-citizens. Cities such as Calgary, for example, have adopted a large-scale vision and planning process for greenspace development, working with citizen groups over many years to develop open space, biodiversity, and park plans. In times of fiscal constraint, many cities have adopted naturalization or volunteer programs as part of their greenspace policies, thereby reducing maintenance costs while giving residents and community partners partial control over the development and management of gardens, parks and planting programs.

## 3. Materials and Methods

### 3.1. Study Area

**Figure 2:**
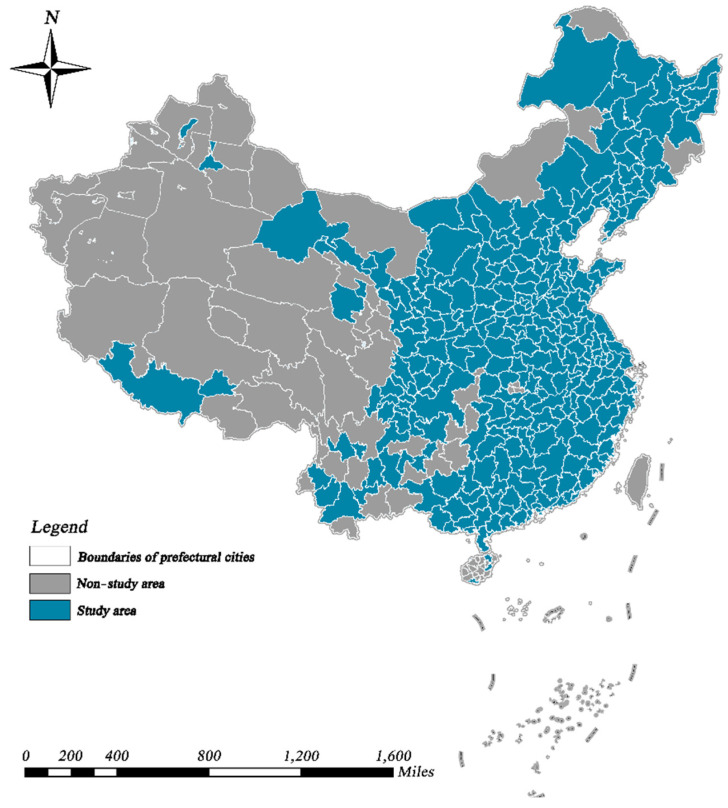
Research areas considered in this study.

China’s unprecedented urbanization has made it an integrated system with complex urban–human–environment interactions, which makes it a perfect target for cross-system sustainability assessments [61,66]. Therefore, in this paper, we assess the coupled and coordinated relationships between urban economic growth and urban greening, taking China as an example. Specifically, the period 2005–2019 was chosen for this study. During this period, China began to shift from a one-sided pursuit of high economic growth to a comprehensive promotion of sustainable development [67]. After excluding cities with unavailable data, we collected a total of 286 cities in China for the study, including four central municipalities, 15 sub-provincial cities, and 267 prefecture-level cities. Hong Kong, Macau, Taiwan and cities in remote western regions were not considered, as shown in Figure 2.

### 3.2. Datasets

Globally, there are various indicators that can be used to quantify urban greening at different scales in different countries [68]. Unlike North America and some countries in Southeast Asia, China has chosen to measure urban greening using indices such as urban green coverage, green space per capita, and urban green space ratio, as these are key indicators for the planning and managing of urban green space systems, as well as the variety of green space development. Therefore, in this paper, we use the public green space per capita to reflect the level of urban greening. The ratio of public urban green space to the number of urban residents is known as public green space per capita, which is one of the most significant metrics for determining the quality of the urban environment. In addition, we use the GDP as a proxy variable for urban economic growth. All of the above data were taken from the China Statistical Yearbook.

### 3.3. Methods

#### 3.3.1. Time–Series Clustering (TSC) Method

To explore the associated spatio-temporal patterns, the TSC approach was used to assess the long time-series changes in CCD, GDP, and urban greening. TSC has been widely employed in a variety of fields, including climate, biology, and geography [69]. Among TSC methods, *k*-means has a clear advantage, due to its effective, stable, and efficient characteristics. In *k*-means analysis, the input data are normalized, in order to make them comparable. The *k*-means algorithm then divides the original dataset into multiple categories, with respect to the similarity of the time-varying patterns. In this process, the center of mass of each cluster changes until the Euclidean distance between the elements of the clusters and the center of mass is minimized to select the optimal number of clusters, and the most reasonable number of clusters is determined when the Sum of Squared Errors (SSE) is sufficiently low and the variation stabilizes [69,70].

#### 3.3.2. CCD Assessment

CCD models have become popular in recent years for the study of cross-system interactions, including for investigation of the coupled coordination between socioeconomic growth and urban environmental quality [40,41]. In particular, the degree of coupling, quantifies the degree of interaction between systems, but does not represent system coordination [43]. As a result, the coupling coordination degree must be determined, in order to represent the coupled system’s shift from disorder to order. The higher the degree of coupling coordination, the better the coordination between the systems, indicating that the two are more capable of developing in tandem [43]. The mathematical formulae for the CCD are calculated as follows:

(1) C=(E×G((E+G)/2)2)12

(2) T=αE+βG (α+β=1)

(3) $$D={\left(E\times G\right)}^{1/2}$$

where **E** and **G** are economy and greening indices, respectively; the degree of connection between the two systems is represented by **C** ∈ [0, 1]; and **D** ∈ [0, 1] is the degree of coupling coordination. The influence of the development state of the two systems on the CCD is represented by **T**, where the contributions of the two systems are represented by **α** and **β**, respectively. In previous studies, both parameters were defined subjectively, and **α** = **β** = 0.5 [41,70]. However, subjective assignments often lead to discrepancies in the assessment of the CCD, so we used an improved method to better assign weights to the above two parameters; that is less-developed systems were given higher values of the contribution coefficients [42]. The specific parameter settings are detailed in Equations (4) and (5):

(4) α=E/(E+G)

(5) β=G/(E+G)

In light of previous research [42,70], a multi-level division criterion was employed to analyze the coordination between urban economic growth and urban greening, as presented in Table 1. Three core phases, four secondary stages, and twelve tertiary stages are included in this evaluation system, with the coordination degree ranging from uncoordinated to coordinated. We go over this scheme in further depth later in the study.

#### 3.3.3. Gini-Based Inequality Assessment

The Gini coefficient was used to assess the degree of disparity between areas in the CCD analysis. This is a well-known approach for determining inequality [45]. Based on the characteristics of the data in the study, the Gini coefficient for CCD was calculated by correcting the equation proposed in [37]. The specific formula is as follows:

(6) $$Gini=1-2\times \overset{n}{\underset{i=1}{\sum }}\overset{i}{\underset{i=1}{\sum }}g_{i}/n\times \overset{i}{\underset{i=1}{\sum }}g_{i}$$

where ***n*** is the number of cities included in the analysis and ***g_i_*** is the CCD of city ***i***. The Gini coefficient ranges from 0 to 1, where a low Gini coefficient indicates that the coupled coordination of the two systems tends to be the same in all cities.

#### 3.3.4. ESDA Method

Moran’s I have been widely utilized to examine spatio-temporal properties through determining the spatial correlations, spatial dependencies, and geographic heterogeneity as a common indicator of ESDA. To assess the spatial correlation and spatial distribution pattern, we used a mixture of the global Moran’s I (***GMI***) and local Moran’s I (***LMI***). The Global Moran’s I (***GMI***) was calculated using the following equation:

(7) $$GMI=\frac{n}{\sum_{i=1}^{n}\sum_{i\neq j}^{n}W_{ij}}\times \frac{\sum_{i=1}^{n}\sum_{i\neq j}^{n}W_{ij}\left(x_{i}-x^{*}\right)\left(x_{j}-x^{*}\right)}{\sum_{i=1}^{n}{\left(x_{i}-x^{*}\right)}^{2}}$$

where $x_{i}$ denotes the relative value of CCD in city i, $x^{*}$ denotes the mean value of x, n stands for the number of cities, and $W_{ij}$ denotes the spatial weight matrix. ***W_ij_*** is the spatial weight matrix defining the structure of the neighborhood, where ***W_ij_*** = 1 if spatial units ***i*** and ***j*** share a border and ***W_ij_*** = 0 otherwise.

The global Moran’s I can reflect spatial autocorrelation, but does not identify the location and type of spatial clusters [71]. Therefore, the Local Moran’s I can be used to determine the local differences and similarities across cities in close proximity. The equation for calculating the Local Moran’s I (***LMI***) is as follows:

(8) $$LMI=\frac{\left(x_{i}-x^{*}\right)}{\frac{1}{n}\sum_{i=1}^{n}\left(x_{i}-x^{*}\right)}\overset{n}{\underset{i=1}{\sum }}\overset{n}{\underset{i\neq j}{\sum }}w_{ij}\frac{1}{n}\overset{n}{\underset{i=1}{\sum }}\left(x_{i}-x^{*}\right)$$

Four outcomes can be obtained when using the Local Moran’s I to identify clusters/outliers: high–high cluster (HH), high–low cluster (HL), low–high cluster (LH), and low–low cluster (LL). Positive spatial correlation is represented by HH and LL, whereas negative spatial correlation is represented by HL and LH.

## 4. Results

### 4.1. Analysis of the Economy and Urban Greening Systems

**Figure 3:**
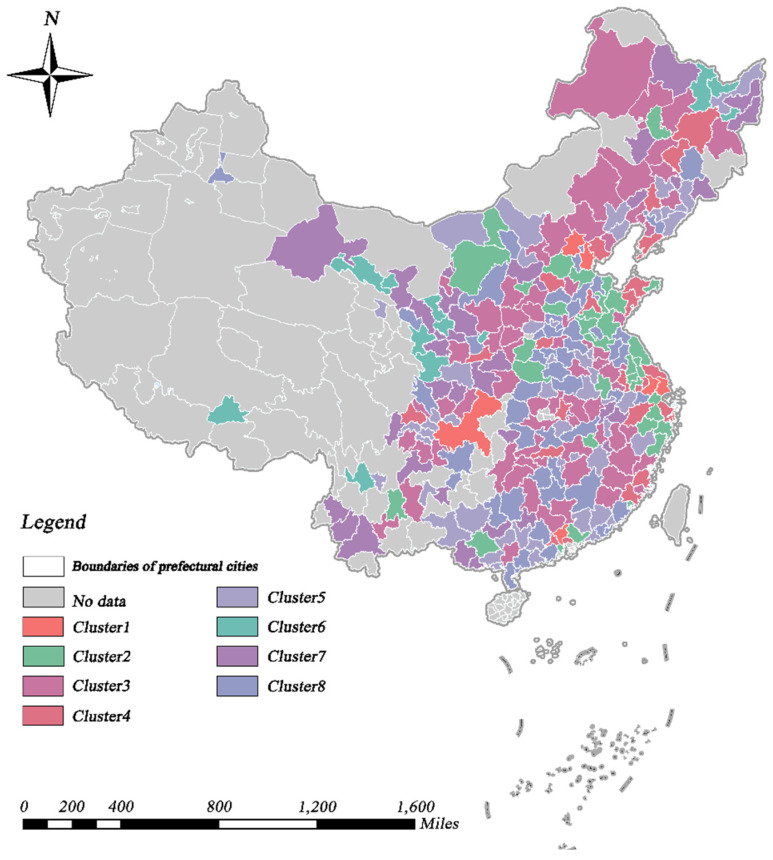
Spatial distribution of the time–series clusters of GDP.

**Figure 4:**
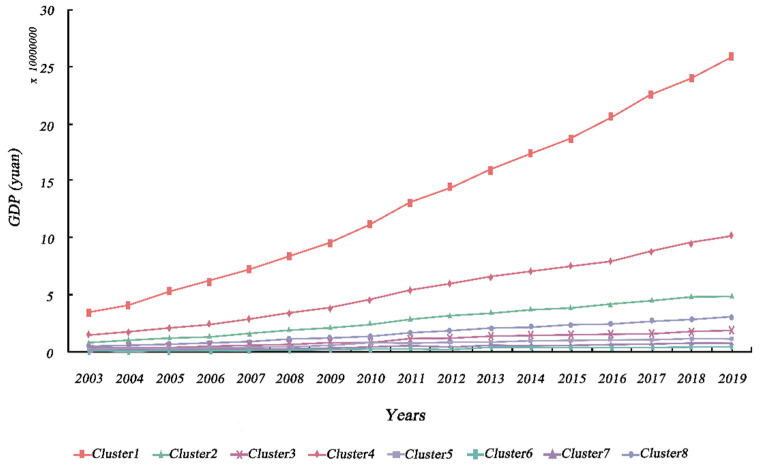
Temporal variation trends of the time-series clusters of GDP.

Based on the TSC results, we analyzed the spatio-temporal patterns of urban economy and urban greening. Specifically, urban economy and urban greening were classified into eight and nine different geographic time-series clusters, respectively, in accordance with the analytical approach described in Section 3.3.1. In terms of GDP, the eight clusters presented substantial regional variability. Likewise, the calculation of annual means for all cities in each cluster, in order to describe the temporal trends, presented significant differences between clusters, as shown in Figure 3 and Figure 4. Cities in eastern coastal regions tended to have larger economic aggregates and more pronounced growth trends than other cities. The key causes for this unequal spatial growth were that the cities described above acted as pioneers, in terms of opening their markets to foreign investors and adopting a free economy after the reform and opening [72]. In addition, hinterland cities, such as Chongqing (municipalities), Wuhan (sub-provincial cities), and Xi’an (regional center cities), which possess a higher urban hierarchy, experienced equally pronounced economic growth, which is partly attributable to the effect of national policies.

**Figure 5:**
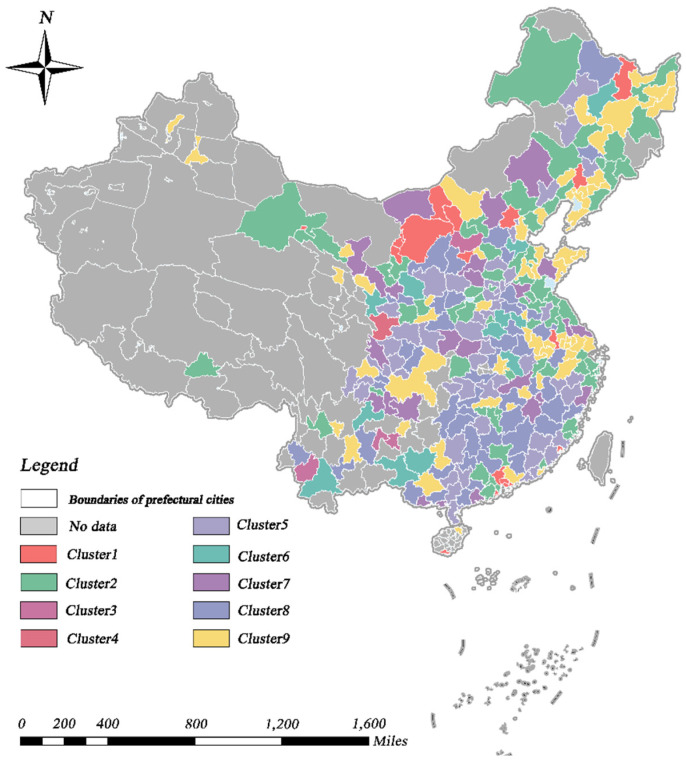
Spatial distribution of the time–series clusters of UPGS.

**Figure 6:**
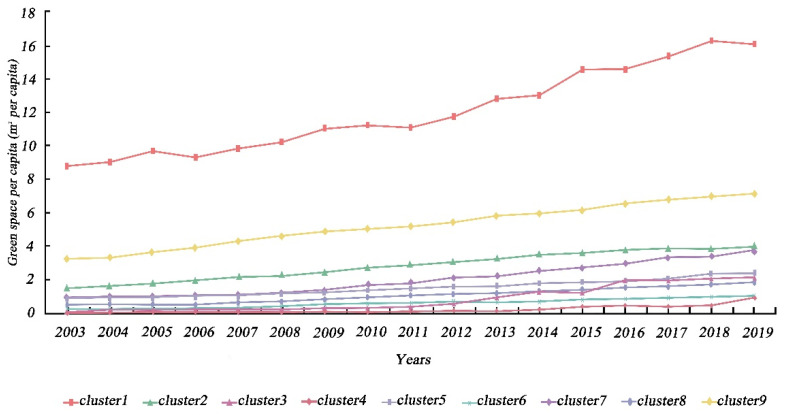
Temporal variation trends of the time-series clusters of UPGS.

As illustrated in Figure 5 and Figure 6, the regional distribution and temporal trends of UPGS clusters presented comparable dynamic features to the GDP clusters. Overall, all cities showed an upward trend in UPGSs, indicating that the public green space per capita was increasing in all cities. In terms of geographic scope, larger annual average UPGSs tended to occur in cities in northern China, as well as in cities along the southeast coast (e.g., Shenyang and its surrounding cities), and some cities in the Shandong Peninsula, Yangtze River Delta, and Pearl River Delta cities consistent with prior research findings [59,73]. In terms of horizon, cluster 1 had the fastest increase in the value of public green space per capita and growth rate, almost doubling 8.7 m^2^/person in 2003 to 16.2 m^2^/person in 2019. Clusters 2, 7, and 9 also presented a more pronounced trend, while the UPGS of the other clusters, although also increasing, do not change significantly over time. In particular, despite the massive influx of immigrant population, Beijing’s public green space per capita rose from 7.48 m^2^/person in 2005 to 16.32 m^2^/person in 2019, further highlighting the central government’s relentless efforts to pursue sustainable regional development and create high-quality living ecological spaces. Similar results were observed in Dongguan, Guangzhou, Jiayuguan, Yinchuan, Zhuhai, and Shizuishan.

### 4.2. The Spatio-Temporal Characteristics of CCD

**Figure 7:**
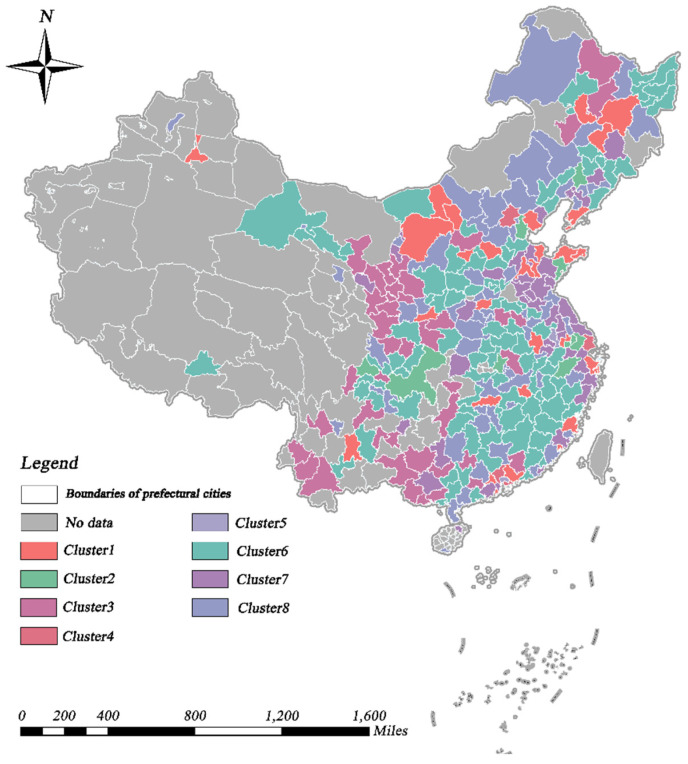
Spatial distribution of the time-series clusters of CCD.

**Figure 8:**
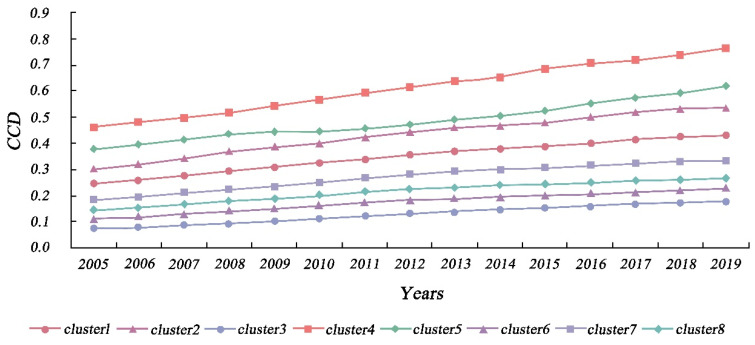
Temporal variation trends of the time-series clusters of CCD.

**Figure 9:**
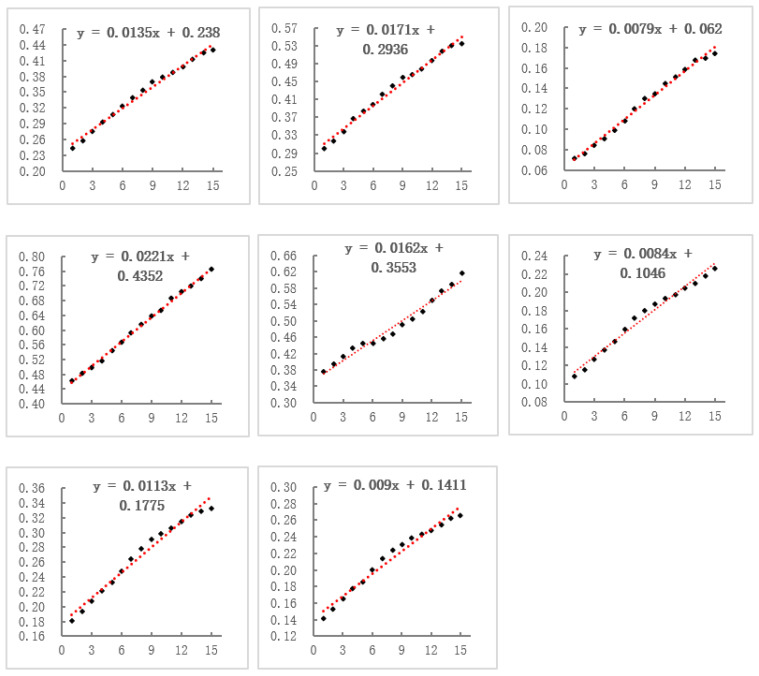
Linear fitting results for CCD values in the eight clusters.

Through TSC analysis, we investigated the spatial and temporal distribution characteristics of the CCD values. Specifically, according to the method mentioned in Section 3.3.1, we evaluated the range of values for the number of clusters (K) and computed the associated SSE values, which were generally low and consistent when K = 8. Therefore, the research region was separated into eight time-series clusters, as shown in Figure 7 and Figure 8. As shown in Figure 9 and Table 2, we first investigated the temporal and spatial evolution patterns of the eight clusters, as well as more specific characteristics of the clusters, such as the number of cities in each cluster, the spatial dispersion pattern, the phase of coupling coordination, and the linear fit of CCD changes. Overall, the regional CCD distribution demonstrated heterogeneity, with the CCD values of eastern coastal cities and inland central cities being much greater than those of other cities, reflecting the more coordinated and balanced growth of the aforementioned cities. Eastern coastal cities and inland central cities show better economic development and have more available funds for environmental protection [6], and citizens are more concerned and involved in local environmental improvement [74]. As a result, these cities are able to ensure economic growth while actively improving urban greening, thus allowing for balanced development of the two systems. It was found that the CCD values of all clusters showed an increasing trend, indicating that the coupling of economic development and urban greening in all cities has become more coordinated over time; however, but the heterogeneity among clusters was still obvious. The highest CCD was that of cluster 4, which has risen from the basic coordination stage in 2005 to the coordination stage in 2019, followed by those of clusters 1, 2, and 5, all rising from a low-level coordination stage in 2005 to a basic coordination stage in 2019. Although the CCD of cluster 3 was also increasing, it was still in the uncoordinated development stage. Clusters 6–8, on the other hand, progressed from the initial uncoordinated stage to the low-level coordinated stage.

**Figure 10:**
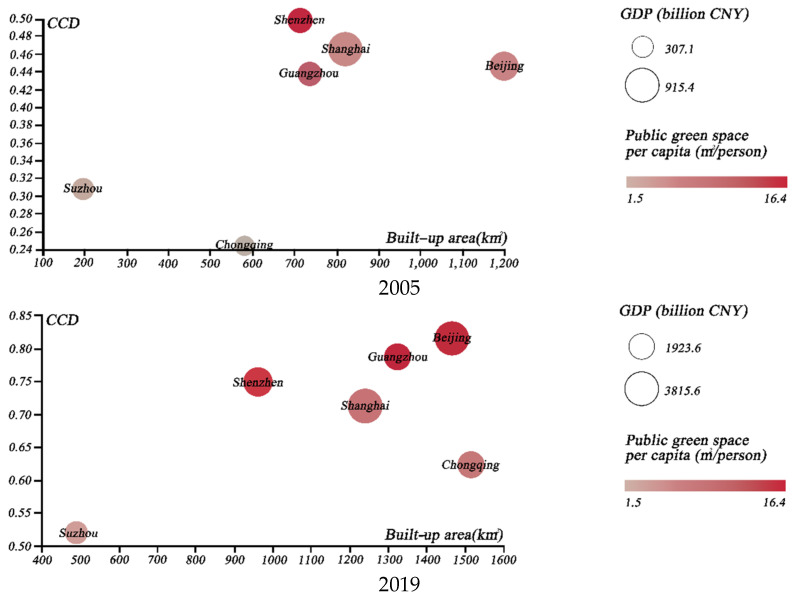
Four–dimensional scatterplot for six cities with the highest GDP levels in 2005 and 2019.

**Figure 11:**
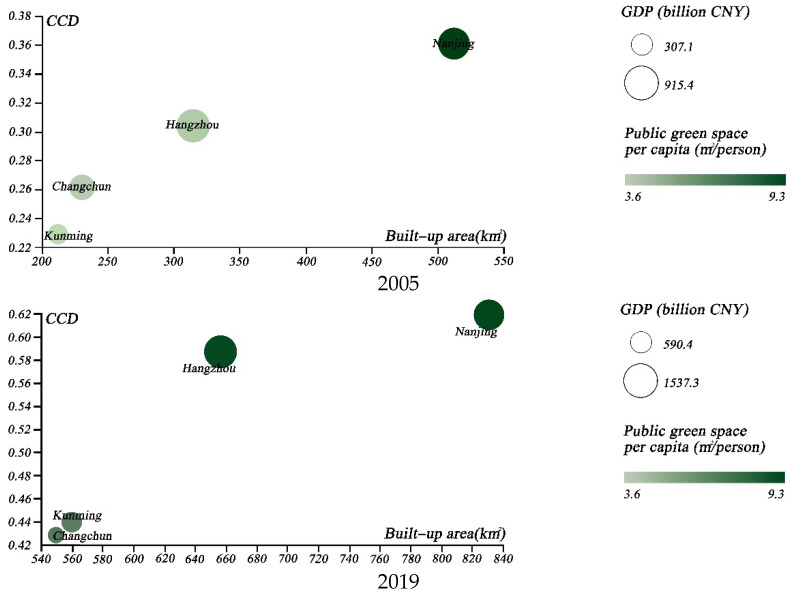
Four-dimensional scatterplot of the four national garden cities in 2005 and 2019.

**Figure 12:**
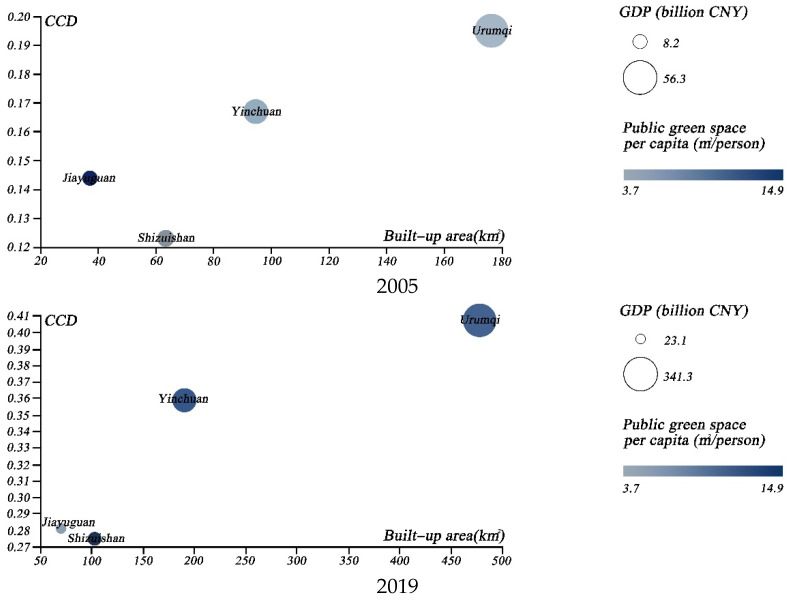
Four-dimensional scatterplot of four central and western cities with high greening levels in 2005 and 2019.

We then chose several specific groups of cities, in order to illustrate the coupled development of urban greening and the urban economy. First, we selected the cities with the highest GDP levels; namely, Beijing, Shanghai, Guangzhou, Shenzhen, Chongqing, and Suzhou. The temporal evolution of the above cities from 2005 to 2019 was compared through the use of scatter plots (Figure 10), in terms of CCD, GDP, public green space per capita, and total built-up area. The results indicated that Beijing surpassed Shenzhen as the most coordinated city. Even with the second largest built-up area of the city, it achieved a significant coordination boost, which can be largely attributed to urban greening management [13]. Secondly, we further analyzed the coupled coordination of the four major Chinese garden cities; namely, Nanjing, Changchun, Hangzhou, and Kunming (Figure 11). Our findings suggest that Nanjing has the best coupling coordination, although the gap between Nanjing and Hangzhou is reducing. Finally, we considered the CCD of four central and western cities with high per capita public green space; namely, Urumqi, Yinchuan, Jiayuguan and Shizuishan (Figure 12). The results indicated that the coupled coordination results for all of these cities were steadily increasing. Except for Urumqi, the other cities were still in the early stages of coordination, and the slow expansion of their urban economies affected the coupled and coordinated development of the two systems. As a result, future policy formulation must take city-specific features.

**Figure 13:**
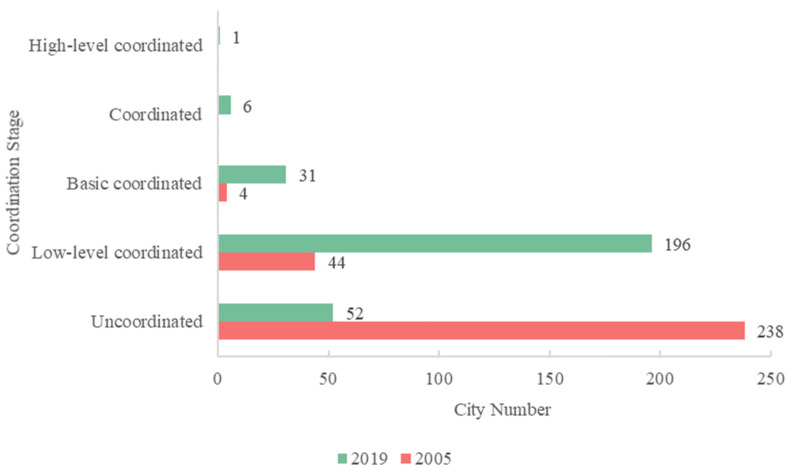
Number of cities in different stages of coupling coordination in 2005 and 2019.

Most cities still have a poor level of coordination, even though economic growth and urban greening cooperation increased between 2005 and 2019 (Figure 13). In fact, the coordination between urban greening and urban economic growth has not received enough attention in other development studies, as urban greening is not the primary issue of environmental protection, compared to previous climate action and sustainable development goals or the current “carbon neutrality, carbon peaking” strategy. The role of urban greening is often overlooked, even though it can enhance the protection and improvement of the urban environment by regulating the microclimate to mitigate the urban heat island effect, absorbing carbon dioxide, and improving the local air environment. Meanwhile, the development and implementation of policies for urban greening development started late in China. Table 3 summarizes the major policies, regulations, and documents related to urban greening issued in mainland China from 2000 to 2019. The most important guiding documents are the Opinions on Further Strengthening Urban Planning and Construction Management, issued by the State Council in 2016, and the Circular on Strengthening Urban Greening Construction, issued in 2020, the former of which sets out clear requirements for the layout, targets, and development goals of urban greening, while the latter reinforces the importance that governments at all levels attach to urban greening work. Therefore, considering the lag of policy and document implementation, the urban economy and urban greening are expected to become more coupled and coordinated in the coming period. Shanghai has accomplished such coupling and development, through the implementation of a series of policies by the Shanghai government. In Shanghai’s urban development, the significance of public green space has been highlighted since the 1980s, shifting from seeing green space as a land-use type to seeing greening as an integrated system that supports urban activities. Green areas are gaining popularity as a guiding principle for future development. As a result, Shanghai is also being planned and developed into a more sustainable ecological city.

**Figure 14:**
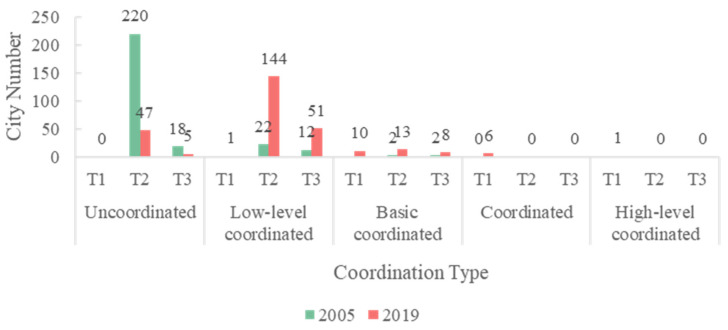
Number of cities in coupling coordination stages T1, T2, and T3 in 2005 and 2019.

**Figure 15:**
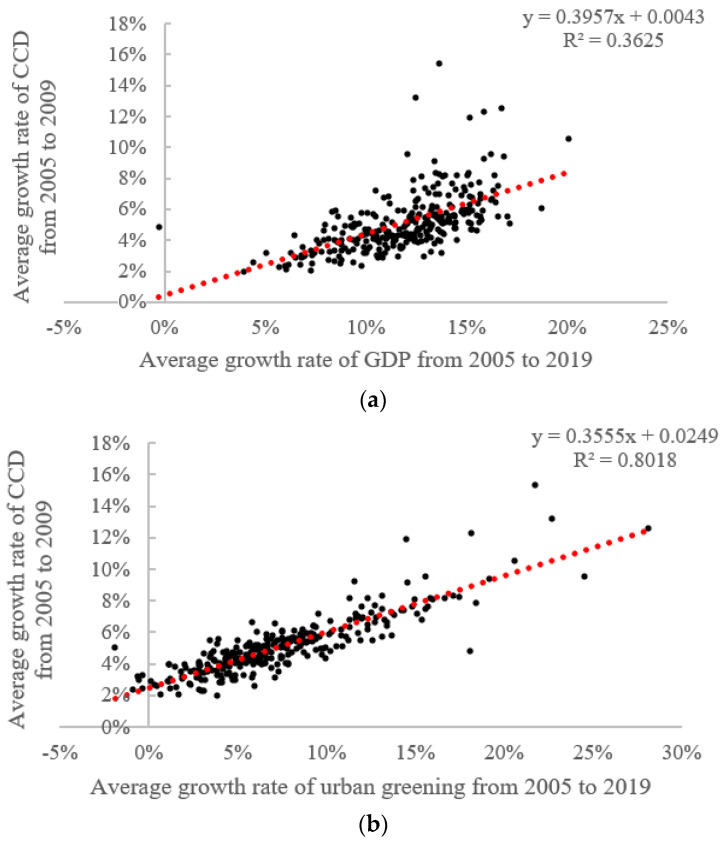
Scatterplot and fitting results of the relationship between the change rate of GDP and CCD (**a**), the change rate of urban greening and CCD (**b**).

On the basis of Figure 13, we further summarize the city types in different coordination stages in Figure 14, where T1 indicates that urban greening lags behind the urban economy, T2 indicates that the above two systems are in balance, and T3 indicates that the urban economy lags behind urban greening. We can observe that, in 2005, more than 90% of the cities in the uncoordinated category reached a balance of the two systems, while the other cities exhibited economic development lagging behind urban greening—a phenomenon also present in cities in the low-level coordination category. By 2019, however, 144 cities had reached a balance between the two systems in the low-level coordination category, while 47 cities reached a balance in the uncoordinated category, highlighting the efforts of local governments in improving the human environment and achieving sustainable regional development. It is worth noting that economic development lagged behind urban greening in 51 cities. Most of these 51 cities, most of which were small cities in the hinterland regions, such as Jiayuguan, were located in the Gobi Desert in the northwest. As the city with the most obvious changes in greening, Jiayuguan ranked first in the country, with 37.91 m^2^ of park green space per capita in 2019—2.7 times the national average for the same period. However, due to its remote location, the natural environment and population mobility limit the inflow of capital, resulting in lagged economic growth. Similar cities included Yinchuan, Lanzhou, and Xining. We then fit the relationships between the change in the CCD, economy, and greening, as shown in Figure 15. The fitting R^2^ value between the greening change rate and CCD change rate reached 0.8018, much better than the fitting result between economy and CCD (0.3625), which emphasizes the need for cities with poor coupling coordination to enhance the coupled and coordinated development of those two systems by strengthening their urban greening layout.

### 4.3. Evolution of Inequality and Spatial Aggregation Patterns of CCD

**Figure 16:**
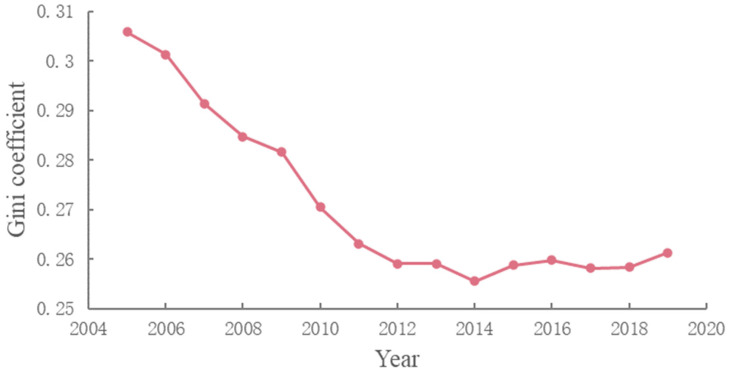
Gini coefficient of CCD between 2005 and 2019.

From 2005 to 2019, the computed Gini coefficient indicated the progression of CCD regional inequality in all cities. This finding is in line with that shown in Figure 16. The evolution of the Gini coefficient for CCD fluctuated between 0.26 and 0.3, with a mean value of 0.271. This finding is not difficult to understand, as the regional inequality of economic development [45,75] and urban greening [34,37,38,48] in China has been widely verified. In terms of temporal evolution, the Gini coefficient presented an overall decreasing trend, indicating improvement in the gap between regions, in terms of the coordinated development status in both economic growth and urban greening.

**Figure 17:**
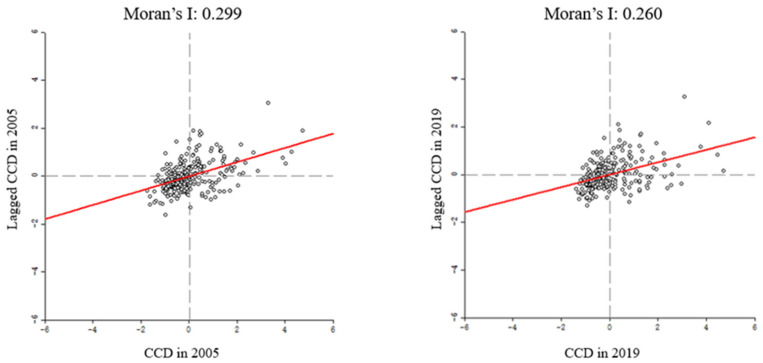
Global Moran’s I scatterplots for CCD in 2005 (**left**) and 2019 (**right**).

The Global Moran’s I of CCD values in 2005 and 2019 were positive, as illustrated in Figure 17, and passed the Z-significance test, which indicates that the CCD value presented significant global spatial aggregation characteristics. Cities with high or low CCD values showed equally high or low values of CCD in neighboring cities. On one hand, this trend might be due to the spillover effect of regional economic growth [44] or the competition between neighboring cities to respond positively to the greening layout. On the other hand, local governments may be more proactive than neighboring cities to enhance the landscape infrastructure and environment, in order to attract more investment and economic activities [76]. We also found a decreasing trend in the GMI for the CCD in 2019, indicating inconsistent attitudes towards the implementation of greening layouts in neighboring cities under the backdrop of broader economic expansion.

**Figure 18:**
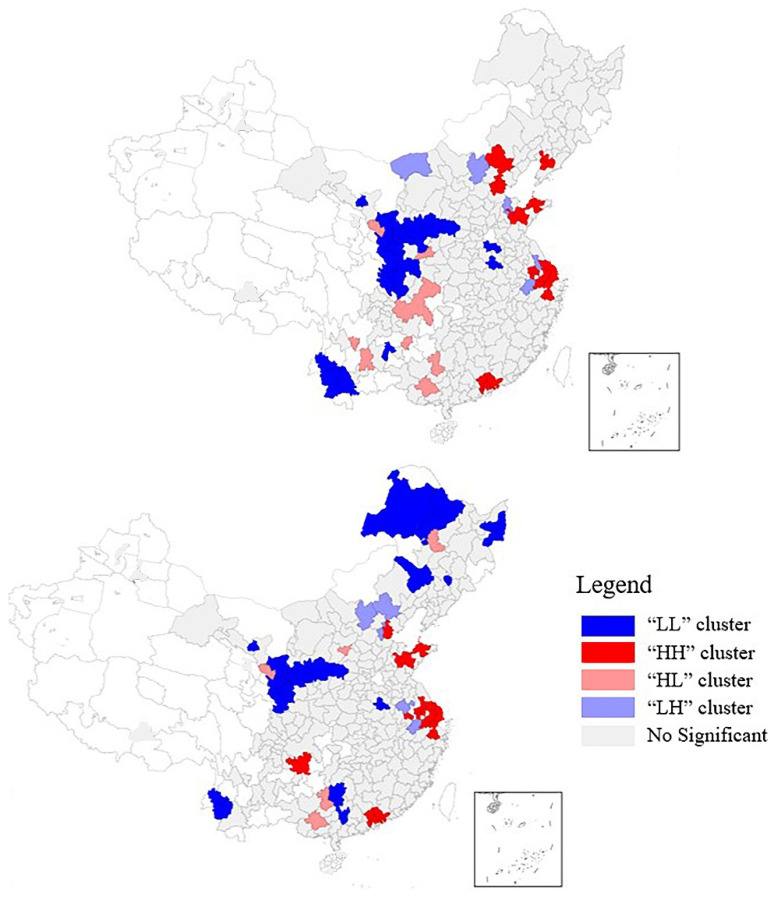
Local Moran scatterplot for CCD in 2005 (**up**) and 2019 (**down**).

Next, we looked at the local geographic features of the CCD value in 2005 and 2019. The results (Figure 18) indicated that most cities did not present substantial local spatial correlation, suggesting that the cities had limited spatial dependency. The cities with typical spatial characteristics were low–low clusters (LL) and high–high clusters (HH). Specifically, the cities in the “HH” and “LL” clusters remained almost identical in number, with some spatial variation. The eastern coastal region was characterized by “HH” clusters, which are reasonably well-coordinated and coupled, and, so, are at the forefront of economic prosperity and greening layout. Cities in the “LL” cluster shifted more noticeably in 2005, mostly in the hinterland regions; meanwhile, the number of “LL” cities in the central region decreased and that in the northeastern region increased significantly in 2019, marking the weakening and strengthening of the spatial correlation between the above two regions, respectively.

### 4.4. Policy Recommendations

A comprehensive and effective policy structure that supports the coordinated and linked development of both considered systems is required, in order to realize the objective of more sustainable cities and communities in the context of rising urbanization. As shown in Figure 13, Figure 14 and Figure 15, although the CCD values between the two systems presented significant improvement, most cities were still characterized by a low-level of coupled and coordinated development, where the economic subsystem tend to lag behind the greening subsystem. However, without effective policies, economic growth has only a short-term effect on the development of urban greenery, rather than the long-term stability of urban ecosystems [76]. Therefore, we systematically outline development proposals involving multiple subjects in the following.

First, economic development should remain as a top priority, especially for cities in the central and western regions. Local governments have used tax policies and low-cost industrial property to stimulate economic growth and income for a long time [77,78]; however, this approach is not sustainable. With freer market intervention, Chinese cities are more apt to shift their green space to other types of land in order to produce more real profits—a tendency that is accelerating [79]. Cities in industrialized nations seek to create or preserve urban green space, in order to make the city more sustainable and habitable, attract more capital and a highly qualified workforce, and improve urban competitiveness [80,81]. Central and western cities should shift their economic development strategies in order to preserve their own greening advantages, focusing on constructing an industrial structure with green production methods, high technological content, low resource consumption, and low pollution, thus promoting green industrial upgrading and establishing a low-carbon cycle development industrial system.

Second, enhancing the urban environment should not be neglected as well. The provision of landscape facilities, such as public green spaces has been widely demonstrated as having a promoting effect on regional economic prosperity. When evaluating local officials for political advancement, as representatives of the people working to promote human well-being, the central government should put greater weight on environmental performance. This is because political promotion is the most practical way to regulate the behavior of local officials in China [82,83]. In pursuit of the goal of sustainable development, urban governance and planning should moreover shift from a crude growth model to a content-based growth model, reversing the situation where economic performance under the leadership of local officials has been dominant in political promotion. In addition, strategic greening initiatives should be addressed in urban planning at the highest levels. Green development plays a vital role in preserving the balance between environmental protection and socio-economic growth. The government should utilize their control of the land supply to encourage urban greening and prioritize eco-city development. Only within such a policy framework can urban green space development be guided to avoid its further loss in the urbanization process.

Third, as the major body in charge of urban operations and administration, local governments play a critical role in fostering long-term regional development. City managers should strengthen their understanding of the advantages of urban greening in shaping urban competitiveness. As Godschalk [84] has proposed, cities are a complex, massive system that cover multi-dimensional values, such as economy, ecology, equity, livability,, etc. Several of these dimensions interact, and a deterioration in any one of them will result in an overall decline in municipal competitiveness. Meanwhile, municipal policymakers should have a long-term plan in place and avoid being blinkered; for example, local governments are more inclined to imitate neighboring cities and use the supply of urban green space as a tool to fuel the real estate market and urban expansion, rather than to truly build a sustainable and livable city [67,85]. In addition, local governments should actively cooperate with non-profit organizations. At present, a certain number of non-profit organizations exist, but they typically do not concentrate on urban greening or urban ecology. However, non-profit groups have been a powerful influence in the building of green space in Western countries [86,87]. This not only reduces government financial pressure, but can also improve government-led urban design and management. As Pincetl [88] has stated, the active participation of non-profits involves a shift in urban governance structures, as they are more capable of capturing the influences of cultural and civil society. Chinese cities have very high potential, in this regard.

### 4.5. Contributions and Limitations

This study innovatively integrates urban greening into a cross-system sustainability assessment framework, complementing research on sustainable cities and communities. Unlike prior research on urban greening, in this study, we consider 286 Chinese cities in our investigation of the coupled coordination of economic growth and urban greening layout, as well as the spatio-temporal patterns of coordination between the two systems, in order to determine whether economic growth promotes urban greening. The results show that our approach effectively captured the interactions between these two aspects. In particular, we found that the more affluent cities in the eastern region performed well in terms of greening and, thus, the coupled coordination degree was higher in these cities. In addition, we observed a decreasing trend of CCD regional inequality year by year, as well as spatial aggregation characteristics. Finally, we proposed policy recommendations in different aspects. Overall, the findings presented herein can help us to better understand the relationships between the economy, urbanization, and the environment, in order to build a more sustainable society in the future.

Of course, this study also had some limitations, which may point out future research priorities. First, the indicators of urban greening in this study were obtained from officially published statistical yearbook data, which has become the most common data source used by researchers, due to ease of accessibility, high accuracy, and large capacity. However, recent studies have pointed out that quantitatively oriented indicators may suffer from inefficiency [73,89]; for example, the spatial distribution of UPGSs based on quantitative analysis may be skewed, as some Chinese cities have local policies that encourage the transfer of planned green space to the suburbs, leaving room for more lucrative real estate development [80,90,91]. Although the quantitative urban greening layout is up to standard, there exists serious variability in spatial planning. This further emphasizes the importance of introducing spatially explicit indicators in future studies. Second, while using a single index as a proxy for urban greening and urban economy without loss of generalizability allows for better cross-regional and national comparisons, a composite index may provide a more detailed description [6,68].

## 5. Conclusions

Using 286 cities in China as sample, we explored the coordination relationships between economic growth and urban greening. The key findings of this study are as follows: (1) economic growth and urban greening were classified into eight and nine clusters, respectively, based on their spatio-temporal heterogeneity. All the clusters within the two systems presented multi-level growth trends. Cities in the eastern coastal region were significantly better than inland regions, in terms of economic growth and greening layout; and (2) eight clusters were identified, in terms of the CCD between economic growth and urban greening. Specifically, the CCD categories ranged from geographically uncoordinated to coordinated. The CCD values of cities in the eastern coastal region were generally higher than in the central and western regions, demonstrating that higher-income regions have better-coordinated development. From 2005 to 2019, the CCD values of all cities presented an increasing trend (the degree of coupled coordination improved). However, more than 60% of cities were still in the stage of uncoordinated or low-level coordinated development; and (3) spatial inequality and agglomeration features of the CCD were seen in all years, although with a downward tendency in all of them across the research period. Our findings emphasized the importance and regional differences of urban greening on one hand, and suggest that urban economic growth has not really brought about improvements in urban greening on the other, and that the urgency of urban greening enhancement remains an important issue for local governments. Based on our findings, we proposed policy recommendations to enhance the pursuit of a friendly and livable urban environment. Of course, our study not only provides an empirical analysis of realistic environmental economic problems in China and complements relevant research gaps in the field of environmental economics, but also lays the foundation for our future research on urban economy and urban environment in the pursuit of sustainable development goals.

## Figures and Tables

**Table 1:** Distribution of developmental phases by CCD.

First tier		Second Tier	Third Tier
Uncoordinated	0 ≤ CCD ≤ 0.2	Uncoordinated	0 ≤ |E-G| ≤ 0.1
			E-G ≥ 0.1
			G-E ≥ 0.1
Transition period	0.2 < CCD ≤ 0.4	Low-level coordinated	0 ≤ |E-G| ≤ 0.1
			E-G ≥ 0.1
			G-E ≥ 0.1
	0.4 < CCD ≤ 0.6	Basic coordinated	0 ≤ |E-G| ≤ 0.1
			E-G ≥ 0.1
			G-E ≥ 0.1
Coordinated	0.6 < CCD ≤ 0.8	Coordinated	0 ≤ |E-G| ≤ 0.1
			E-G ≥ 0.1
			G-E ≥ 0.1
	0.8 < CCD ≤ 1.0	High-level coordinated	0 ≤ |E-G| ≤ 0.1
			E-G ≥ 0.1
			G-E ≥ 0.1

Note: E-G ≥ 0.1 indicates that greening lags behind the economy. G-E ≥ 0.1 indicates that economy lags behind the greening.

**Table 2:** Properties of the eight CCD clusters.

Cluster	Count of Cities	Pattern of Spatial Dispersion	Temporal Status Evolution
1	29	Small and medium-sized cities in the eastern coastal region and inland provincial capitals, such as Changsha, Xi’an, and Zhengzhou.	Rose from the low-level coordination stage to the basic coordination stage.
2	9	Scattered distribution, mainly in sub-provincial cities.	Rose from the low-level coordination stage to the basic coordination stage.
3	41	Small and medium-sized cities, mainly in the central and western regions.	Remained at the uncoordinated development stage.
4	4	Beijing, Shanghai, Guangzhou, and Shenzhen.	Rose from the basic coordination stage to the coordination stage.
5	2	Dongguan and Nanjing.	Rose from the low-level coordination stage to the coordination stage.
6	95	The most widely distributed, covering a large number of small and medium-sized cities in the northeast and central regions.	Remained at the uncoordinated development stage.
7	42	Small and medium-sized cities, mainly in the eastern region.	Rose from the uncoordinated stage to the low-level coordination stage.
8	64	Located in clusters throughout the research area.	Rose from the uncoordinated stage to the low-level coordination stage.

**Table 3:** Major policies, regulations, and documents related to urban greening in mainland China from 2000 to 2020.

Year	Name of Policy, Regulations, or Documents	Publishing Departments
2000	The State Council on strengthening urban greening construction notice	State Council
2002	Urban green space classification standards	Ministry of Housing and Urban-Rural Development(MHURD)
2010	Evaluation standards for urban landscape and greening	MHURD
2011	Urban green line management measures	MHURD
2012	Guiding opinions on promoting the healthy development of urban landscaping	MHURD
2016	Several opinions on further strengthening urban planning and construction management	State Council
2016	National urban ecological protection and construction plan	MHURD
2017	Urban green space classification standards	MHURD
2020	Notice on strengthening the construction of urban greening	State Council
2020	Urban greening planning and construction indicators of the provisions	Ministry of Industry and Information Technology

## Data Availability

Publicly archived datasets China Statistical Yearbook on Environment and China Statistical Yearbook 2005–2019 were analyzed in this study. These data can be found at: https://data.cnki.net (accessed on 15 April 2022).
